# Regulatory network of GSK3-like kinases and their role in plant stress response

**DOI:** 10.3389/fpls.2023.1123436

**Published:** 2023-03-01

**Authors:** Yun Song, Ying Wang, Qianqian Yu, Yueying Sun, Jianling Zhang, Jiasui Zhan, Maozhi Ren

**Affiliations:** ^1^ School of Life Sciences, Liaocheng University, Liaocheng, China; ^2^ Institute of Urban Agriculture, Chinese Academy of Agricultural Sciences, Chengdu, China; ^3^ Hainan Yazhou Bay Seed Laboratory, Sanya, China; ^4^ National Nanfan Research Institute (Sanya), Chinese Academy of Agricultural Sciences, Sanya, China; ^5^ Department of Forest Mycology and Plant Pathology, Swedish University of Agricultural Sciences, Uppsala, Sweden

**Keywords:** glycogen synthase kinase 3 (GSK3), plant, hormone, abiotic stress, biotic stress

## Abstract

Glycogen synthase kinase 3 (GSK3) family members are evolutionally conserved Ser/Thr protein kinases in mammals and plants. In plants, the GSK3s function as signaling hubs to integrate the perception and transduction of diverse signals required for plant development. Despite their role in the regulation of plant growth and development, emerging research has shed light on their multilayer function in plant stress responses. Here we review recent advances in the regulatory network of GSK3s and the involvement of GSK3s in plant adaptation to various abiotic and biotic stresses. We also discuss the molecular mechanisms underlying how plants cope with environmental stresses through GSK3s-hormones crosstalk, a pivotal biochemical pathway in plant stress responses. We believe that our overview of the versatile physiological functions of GSK3s and underlined molecular mechanism of GSK3s in plant stress response will not only opens further research on this important topic but also provide opportunities for developing stress-resilient crops through the use of genetic engineering technology.

## 1 Introduction

As a major source of food, fuel, and fiber, plant supports human society and sustains the global ecosystem by photosynthesis (Waadt et al., 2022; Zhang et al., 2022a). Plants are challenged throughout their life cycles by adverse environmental conditions including abiotic stresses such as drought, salinity, extreme temperatures, nutrient deficiency, and toxic metal levels in the soil as well as biotic stresses such as pathogen infection and herbivore attack (Peck and Mittler, 2020). These adverse environmental conditions limit the distribution of plants, threaten their growth, and reduce crop productivity, eventually resulting in devastating impacts on our economy. To reduce the impacts, it is necessary to understand how plants adapt to these adverse environmental conditions. There is growing evidence that plants have evolved sophisticated mechanisms to respond to environmental stresses, with recent results from some model and crop species the involvement of glycogen synthase kinase 3 (GSK3) protein in such adaptation.

## 2 Identification of plant GSK3s

The glycogen synthase kinase GSK3, also known as shaggy-like kinase (SK), was first identified in humans and functions as a regulator of glycogen synthase (Woodgett, 1991; Li et al., 2021). Only two *GSK3* isoforms (i.e., *GSK3α* and *GSK3β*) are present in human genomes. They regulate diverse biochemical and cellular processes (Beurel et al., 2015; Patel and Woodgett, 2017). In contrast to mammalian GSK3s, GSK3-like kinases in plants are encoded by multiple homologs of the genes and are groiped into four subfamilies (**Table 1**) (Li et al., 2021). For example, there are ten and nine GSK3-like kinases in the dicot arabidopsis (*Arabidopsis thaliana*) and the monocot rice (*Oryza sativa*), respectively (Qi et al., 2013; Youn and Kim, 2015). The *GSK3*-like genes have also been identified in several important crop species, such as barley (*Hordeum vulgare*), cotton (*Gossypium hirsutum*), maize (*Zea mays*), pepper (*Capsicum annuum*), potato (*Solanum tuberosum*), sorghum (*Sorghum bicolor*), soybean (*Glycine max*), wheat (*Triticum aestivum*) (**Table 1**) (Chen et al., 2003; Hirano et al., 2017; Qiu et al., 2018; Wang et al., 2018b; Cheng et al., 2020; Kloc et al., 2020; Wang et al., 2020; He et al., 2021; Huang et al., 2021; Hou et al., 2022; Wang et al., 2022; Zolkiewicz and Gruszka, 2022; Zhang et al., 2022b). Multiple copies of *GSK3* in plant genomes indicate that this gene plays important and diverse roles in the evolutionary adaptation and life strategies of plants (Qi et al., 2013; Li et al., 2021). Indeed, despite evolutionary conservation, the specific function of GSK3 in plants can vary among species.

**Table 1:** GSK3s in various eukaryotes.

Plant Species	Subgroup	Gene Name	Function	References
*Arabidopsis thaliana*	I	AtSK11, AtSK12, AtSK13	BR signal/Flower development/ Osmotic stress	Kondo et al., 2014; Tamaki et al., 2020; Li et al., 2018; Chen et al., 2020b; Stampfl et al., 2016; Dong et al., 2020; Dal Santo et al., 2012
	II	AtSK21, AtSK22, AtSK23	BR signal/Growth/Salt stress	Li and Nam, 2002; Yan et al., 2009; Song et al., 2021
	III	AtSK31, AtSK32	BR signal/Flower development/ Osmotic stress	Rozhon et al., 2010; Dong et al., 2015; Eremina et al., 2016
	IV	AtSK41, AtSK42	Osmotic stress	Youn and Kim, 2015
*Oryza sativa*	I	OsSK11, OsSK12, OsSK13	BR signal	Youn and Kim, 2015
	II	OsSK21, OsSK22, OsSK23, OsSK24	BR signal/Growth/Abiotic stress/ Biotic stress	Tong et al., 2012; Youn and Kim, 2015; Yang et al., 2016; Xiao et al., 2017; Gao et al., 2019; Min et al., 2019; Lyu et al., 2020; Xiao et al., 2020; Sun et al., 2015; Sun et al., 2018; Che et al., 2015; Liu et al., 2017; Koh et al., 2007; He et al., 2020
	III	OsSK31	BR signal	Youn and Kim, 2015
	IV	OsSK41	Growth	Xia et al., 2018; Ying et al., 2018; Hu et al., 2018
*Hordeum vulgare*	II	HvGSK1.1	Growth/Salt stress	Kloc et al., 2020
*Gossypium hirsutum*	I	GhSK11, GhSK12, GhSK13, GhSK14	BR signal/Fiber development/Stress response	Wang et al., 2018b; Wang et al., 2020
	II	GhSK21, GhSK22, GhSK23, GhSK24, GhSK25, GhSK26	BR signal/Fiber development/Stress response	Wang et al., 2018b; Song et al., 2021
	III	GhSK31, GhSK32, GhSK33, GhSK34, GhSK35, GhSK36	Fiber development/Stress response	Wang et al., 2018b
	IV	GhSK41, GhSK42, GhSK43, GhSK44	BR signal/Fiber development/Stress response	Wang et al., 2018b; Wang et al., 2020
*Zea mays*	II	ZmSK1, ZmSK2	BR signal/Embryonic development	Wang et al., 2022
*Capsicum annuum*	II	CaSK23	Biotic stress	Qiu et al., 2018
*Solanum tuberosum*	I	StSK11, StSK12, StSK13	BR signal/Abiotic stres	Huang et al., 2021
	II	StSK21, StSK22, StSK23	BR signal/Salt stress	Huang et al., 2021
	III	StSK31, StSK32	BR signal/Abiotic stress	Huang et al., 2021
	IV	StSK41	Abiotic stress	Huang et al., 2021
*Sorghum* *bicolor*	II	SbBIN2	BR signal	Hirano et al., 2017
*Glycine max*	II	GmBIN2	Salt and drought stress	Wang et al., 2018a
	II	GmSK2-8	Legume-rhizobium symbiosis	He et al., 2021
*Triticum aestivum*	I	TaSK11-3A,3B,3D TaSK12-4A,5B,5D TaSK13-1A,1B,1D	Drought and salt stress	Zhang et al., 2022b
	II	TaSK21-3A,3B,3D TaSK22-1A,1B,1D	BR signal/Salt stress	Chen et al., 2003; Cheng et al., 2020; Zhang et al., 2022b
	III	TaSK31-1A,1B,1D	Drought and salt stress	Zhang et al., 2022b
	IV	TaSK41-1A,4A,1B,1D	Drought and salt stress	Zhang et al., 2022b

In plants, the best-known representative GSK3 is brassinosteroid insensitive 2 (BIN2/SK21). It is a key negative regulator of the plant steroid hormones brassinosteroid (BR) response in *Arabidopsis* (Li and Nam, 2002). The *BIN2* gene has two closest homologs i.e., *BIN2-like 1* (*BIL1*) and *BIN2-like 2* (*BIL2*) functioning redundantly with BIN2 to negatively regulate the BR signaling. The *bin2 bil1 bil2* triple mutants showed a constitutive BR-activation phenotype (Yan et al., 2009). The BR signaling has been well elucidated and proved to be an important regulator of plant physiological and biological processes including seed germination, cell division, elongation and differentiation, leaf senescence and response to biotic and abiotic stresses (Rozhon et al., 2010; Kondo et al., 2014; Dong et al., 2015; Anwar et al., 2018; Peres et al., 2019; Ackerman-Lavert and Savaldi-Goldstein, 2020; Tamaki et al., 2020; Zolkiewicz and Gruszka, 2022). BRs are recognized by the leucine-rich repeat receptor-like (LRR-RLK) protein brassinosteroid-insensitive 1 (BRI1) and its co-receptor BRI1 associated receptor kinase 1 (BAK1) at the membrane (Wang et al., 2008; Santiago et al., 2013). Moreover, BRI1 phosphorylates its inhibitor, BRI1 kinase inhibitor 1 (BKI1), guides the dissociation of BKI1 from the membrane, and enables the formation of the BRI1-BAK1 receptor complex to fully initiate the signaling cascade (Wang and Chory, 2006). The active receptor complex phosphorylates downstream proteins, including the BR signaling kinases (BSKs), constitutive differential growth 1 (CDG1) protein, and finally *bri1* suppressor1 phosphatase (BSU1) (Tang et al., 2008; Kim et al., 2011). BSU1 and its homologs then dephosphorylate and inhibit the glycogen synthase kinase 3 kinase BIN2, which is a major repressor of the BR signaling (Kim et al., 2009). Without BR, BIN2 phosphorylates two key transcription factors *bri1*-EMS-suppressor 1 (BES1) and brassinazole-resistant 1 (BZR1), which eventually inhibits the BR downstream signaling (Li and Nam, 2002). Upon initiation of the BR signaling, as for the BIN2 inactivation, BZR1 and BES1 proteins bind to the promoters of the BR-responsive genes (Nolan et al., 2020).

In the monocot model rice, several numbers of *Os*SKs/*Os*GSK3s proteins are also found to function as negative regulators of BR signaling by phosphorylating and regulating the activity of several transcription factors involved in the BR-dependent gene expression (Tong et al., 2012; Zhang et al., 2012; Sun et al., 2015; Yang et al., 2016; Qiao et al., 2017; Xiao et al., 2017; Sun et al., 2018; Xia et al., 2018; Ying et al., 2018; Gao et al., 2019; Min et al., 2019; Lyu et al., 2020; Xiao et al., 2020). For example, *Os*SK22 can phosphorylate and stabilize *Os*PUB24 to promote the degradation of *Os*BZR1, which is similar to what was discovered in *Arabidopsis* (Min et al., 2019). Thus, *Os*SK22 was named as *Os*GSK2 and is widely considered as the rice ortholog of BIN2. In conclusion, the GSK3 proteins function as negative regulators of BR signaling, and their function is generally conserved among dicots and monocots.

## 3 Upstream regulators and downstream substrates of GSK3s

**Figure 1:**
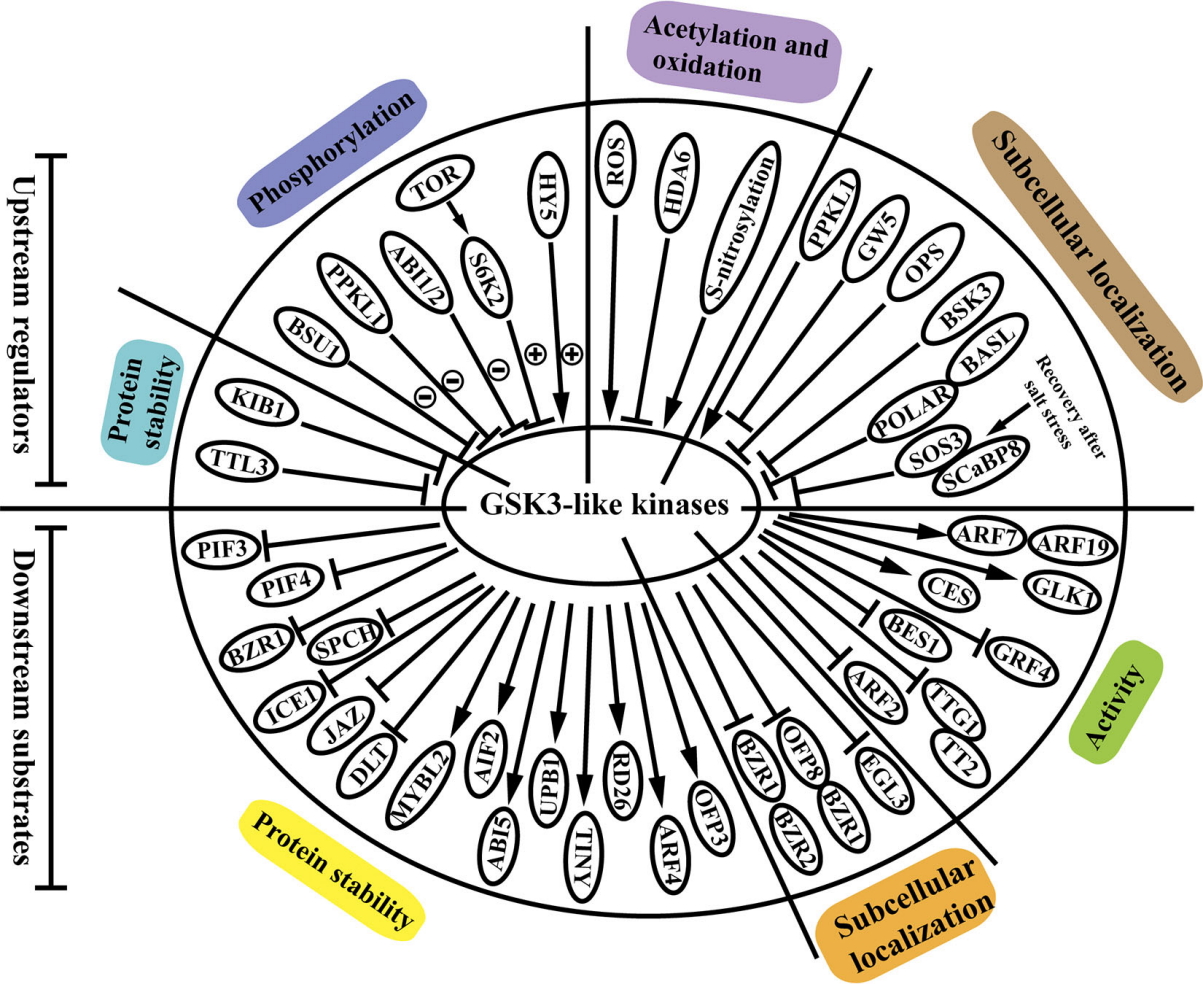
Upstream regulators and downstream substrates of GSK3-like kinases in plants. The GSK3s-like kinases are regulated *via* modulation of protein stability, protein activity *via* various modifications including phosphorylation, acetylation and oxidation, and subcellular localization. Meanwhile, GSK3s-like kinases modulate downstream substrates through phosphorylation, and affects the substrates stability, localization, and activity of these substrates. Circled plus or circled minus signs indicate phosphorylation and dephosphorylation, respectively. Arrows and bar ends indicate stimulatory and inhibitory action, respectively. Abbreviations: TTL3: tetratricopeptide thioredoxin-like 3; KIB1: kink suppressed in *bzr1-1D* 1; BSU1: *bri1* suppressor1 phosphatase; PPKL1: phosphatase with Kelch-like repeat domains; ABI1/2: ABA insensitive 1/2; TOR: target of rapamycin; S6K2: ribosomal protein S6 kinase 2; HY5: elongated hypocotyl 5; ROS: reactive oxygen species; HDA6: histone deacetylase 6; GW5: grain width 5; OPS: octopus; BSK3: brassinosteroid signaling kinase 3; BASL: breaking of asymmetry in the stomatal lineage; POLAR: polar localization during asymmetric division and redistribution; SOS3: salt overly sensitive 3; SCaBP8, SOS3-like calcium binding protein 8; PIF3: phytochrome interacting factor 3; PIF4: phytochrome interacting factor 4; BZR1: brassinazole-resistant 1; SPCH: speechless; ICE1: inducer of CBF expression 1; JAZ: jasmonate ZIM domain; DLT: dwarf and low-tillering; MYBL2: myeloblastosis family transcription factor-like 2; AIF2: ATBS1-interacting factor 2; ABI5: abscisic acid insensitive 5; UPB1: upbeat 1; TINY: AP2 family transcription factor; RD26: NAC transcription factor; ARF4: auxin response factor 4; OFP3: OVATE family protein 3; BZR2: brassinazole-resistant 2; OFP8: OVATE family protein 8; EGL3: enhancer of glabra 3; ARF2: auxin response factor 2; TTG1: transparent testa glabra 1; TT2: transparent testa 2; BES1: *bri1*-EMS-suppressor 1; GRF4: growth regulating factor; CES: cesta; GLK1: golden2-like 1; ARF7: auxin response factor 7; ARF19: auxin response factor 19.

Emerging evidence has shown that GSK3s proteins can perceive upstream signals and be regulated at the post-translational level. Moreover, the downstream targets phosphorylated by the GSK3s proteins have also been extensively elucidated. In this section, we review how the GSK3s proteins are modulated by the known upstream regulators and how they regulate the downstream substrates through phosphorylation (**Figure 1**).

### 3.1 Upstream regulators of GSK3s

Numerous studies have revealed that the GSK3s proteins are mainly regulated at the post-translational level *via* modulation of protein stability, protein activity *via* various modifications (i.e., phosphorylation, acetylation and oxidation), and subcellular localization (Li et al., 2021). A series of GSK3s proteins, such as *At*BIN2, *At*BIL1, *At*BIL2, *At*SK11, *At*SK12, *Os*GSK2, and *Ta*SG-D1, is degradated through proteasome-mediated pathway (Li and Nam, 2002; Peng et al., 2008; Yan et al., 2009; Tong et al., 2012; Li et al., 2018; Cheng et al., 2020; Chen et al., 2020b). Moreover, the proteasome-mediated regulation of GSK3s is modulated *via* a conserved C-terminal TREE motif, and mutations in this motif cause the gain-of-function effects. For example, the mutation in the TREE motif impaired the ubiquitylation-dependent proteasomal degradation of BIN2 mediated by the E3 ubiquitin ligase kink suppressed in *bzr1-1D* 1 (KIB1). Moreover, homologous proteins of KIB1 are also involved in the BR-induced proteasomal degradation of BIN2, and they function redundantly in the suppression of the GSK3 kinase activity (Zhu et al., 2017). But how KIB1 and its homologous proteins interact with other proteins of the BR signaling remains elusive (Li et al., 2021).

Evidence that the activity of GSK3s is altered by protein modifications including phosphorylation, acetylation, and oxidation is also accumulated. For example, dephosphorylation and phosphorylation of GSK3s, which are the most common ways in the regulation of GSK3s (**Figure 1**), significantly change the activities of the proteins in various species. The suppressor of *bri1* (BSU1) dephosphorylates BIN2 at phospho-tyrosine 200, and deactivates BIN2 kinase activity in the BR signaling transduction (Kim et al., 2009). Similarly, the BIN2 ortholog in rice *Os*GSK3 (*Os*SK23) is dephosphorylated by the rice ortholog of BSU1, *Os*PPKL1 (phosphatase with Kelch-like repeat domains), and affectes BR signaling (Gao et al., 2019). However, the effect of *Os*PPKL1 on *Os*GSK3 activity is completely different with that caused by *At*BSU1. The dephosphorylation of *At*BIN2 kinase resulted in the BIN2 degradation, but in rice, *Os*PPKL1 stabilized the *Os*GSK3 protein. The opposite effect on GSK3s protein stabilization may be related with its specific role in different species, which requires further investigation. The bZIP transcription factor, elongated hypocotyl 5 (HY5), binds to BIN2 and enhances the autophosphorylation of BIN2 at the tyrosine 200 residue (Li et al., 2020a). Ribosomal protein S6 kinase 2 (S6K2) phosphorylates BIN2 at serines 187 and 203, and inhibites BIN2 activity in *Arabidopsis* (Xiong et al., 2017). They also find that the phosphorylation of BIN2 depends on the upstream TOR-S6K2 signaling, which plays a vital role in coordinating plant growth and stress responses. Histone deacetylase 6 (HDA6) can also interact with and deacetylate BIN2 at lysine 189 to repress its kinase activity (Hao et al., 2016). The histone acetylation modification of GSK3s proteins is also identified in rice (Hou et al., 2022). The histone deacetylase1 (*Os*HDAC1) directly interactes with and deacetylates *Os*GSK2, and eventually inhibits *Os*GSK2 activity. The above results suggest the conserved regulation mechanism of GSK3 by acetylation in *Arabidopsis* and rice. The expression pattern of *HDA6* gene when exposed to BR is opposite with that of *OsHDAC1* gene, which needs further research. Moreover, it has been found that the interaction of BIN2 and BES1 is oxygen-dependent, and the activity of BIN2 is regulated by the oxidation of several cysteine (Cys) residues including C59, C95, C99, and C162 (Song et al., 2019).

Emerging evidence has indicated that regulation of the subcellular localization of GSK3s significantly influences their biological functions in plants. Plasma membrane recruitment of GSK3s modulates BR signaling in *Arabidopsis* and rice. In *Arabidopsis*, several proteins including octopus (OPS), tetratricopeptide thioredoxin-like 3 (TTL3), and brassinosteroid signaling kinase 3 (BSK3), interact with and recruit BIN2 to the plasma membrane, and prevent BIN2 inhibitory role in the nucleus in the BR signaling pathway. Among these proteins, TTL3 affects BIN2 subcellular localization and promotes the degradation of BIN2 (Anne et al., 2015; Amorim-Silva et al., 2019; Ren et al., 2019). As mentioned above, *Os*PPKL1 dephosphorylated and stabilized *Os*GSK3 in the cytoplasm to modulate BR signaling (Gao et al., 2019). We find that TTL3 not only influences BIN2 stability, but also affects the BIN2 subcellular localization. *Os*PPKL1 not only influences BIN2 through protein modifications, but also alters the BIN2 subcellular localization. These results together indicates that the upstream proteins can regulate GSK3s proteins in more ways than one. In rice, a calmodulin binding protein grain width 5 (GW5) can also physically interact with and recruit *Os*GSK32 to the plasma membrane, thus resulting in accumulation of unphosphorylated *Os*BZR1 and dwarf and low-tillering (DLT) to promote BR signaling (Liu et al., 2017). Moreover, GSK3s subcellular localization mediates plant transition from the salt stress response to growth recovery. BIN2 is mainly locialized in the nucleus to regulate BR signaling and plant growth under salt stresses. In the recovery phase, BIN2 was recruited to the plasma membrane by the salt-triggered calcium sensors salt overly sensitive 3 (SOS3) and SOS3-like calcium binding protein 8 (SCaBP8) to regulate the salt stress response (Li et al., 2020b). Taking the above aspects into account, we can conclude that the altered subcellular localization of GSK3s proteins contributes to realize GSK3s proteins functions in the balance between plant growth and stress response. GSK3s subcellular localization also influences stomatal patterning. Stomatal cell lineage is an archetypal example of asymmetric cell division (ACD). The scaffold membrane protein polar localization during asymmetric division and redistribution (POLAR) can confine BIN2 and *At*SK12 to the cytosol to drive ACD. After ACDs, BIN2 and *At*SK12 phosphorylate and dissociate from POLAR (Houbaert et al., 2018).

### 3.2 Downstream targets of GSK3s

Like upstream regulation, GSK3s also intensively modulate downstream substrates through phosphorylation to accomplish their functions in regulating plant development and stress responses. GSK3s can phosphorylate a series of substrates including transcription factors, cofactors, kinases, scaffold proteins, cytoskeleton proteins, cyclins, metabolic enzymes, and components of the ubiquitin-proteasome system (UPS). The role of GSK3s in modulating proteins including kinases, scaffold proteins, cytoskeleton proteins, cyclins, metabolic enzymes, and components of the ubiquitin-proteasome system (UPS), has been extensively discussed in several excellent recent reviews (Youn and Kim, 2015; Li et al., 2021; Mao et al., 2021; Zolkiewicz and Gruszka, 2022). In this section, we mainly focus on the discoveries about effects of GSK3s on the transcription factors and cofactors, which play essential roles in modulating stress responsive gene expression.

GSK3s-mediated phosphorylation of the transcription factors and cofactors, eventually affects the protein stability, localization, and activity of these substrates. Recent reports have revealed that GSK3-mediated phosphorylation not only promotes the degradation of the substrates but also stabilizes downstream targets. During stomatal patterning, BIN2 and MAPKs phosphorylate and promote the degradation of speechless (SPCH), which is a bHLH transcription factor in the nucleus required for stomatal initiation (Gudesblat et al., 2012). Phosphorylation and degradation of phytochrome interacting factor 3 (PIF3) and 4 (PIF4) are facilitated by BIN2 to control skotomorphogenesis and hypocotyl elongation, respectively (Bernardo-García et al., 2014; Ling et al., 2017). In plants, GSK3s also stabilizes transcription factors through phosphorylation. A series of transcription factors, such as myeloblastosis family transcription factor-like (MYBL2; a MYB transcription factor), ATBS1-interacting factor 2 (AIF2; a bHLH transcription factor), abscisic acid insensitive 5 (ABI5; a bZIP transcription factor), upbeat 1 (UPB1; a bHLH transcription factor), TINY (AP2 family transcription factor) and RD26 (NAC family transcription factor), are phosphorylated and stabilized by BIN2 (Ye et al., 2012; Hu and Yu, 2014; Kim et al., 2017; Jiang et al., 2019; Xie et al., 2019; Li et al., 2020c). However, the underlying molecular mechanisms of substrates degradation or accumulation associated with BIN2-catalyzed phosphorylation have not been investigated.GSK3s-mediated regulation of subcellular localization in substrates is ubiquitous in plants. BIN2-catalyzed phosphorylation of BZR1/BZR2 promotes their binding to the 14-3-3 proteins, resulting in cytoplasmic retention and inhibition of BR-regulated gene expression (Gampala et al., 2007). In rice, phosphorylation of OsBZR1 and transcription factor ovate family protein 8 (OFP8) by OsGSK2 also results in their nuclear export (Yang et al., 2016). Furthermore, GSK3s-mediated phosphorylation can restrict the nuclear localization of their substrates. For example, the bHLH transcription factor enhancer of glabra 3 (EGL3) is phosphorylated by BIN2, thus remaining nuclear localization and participating in the BR-regulated root epidermal cell patterning (Cheng et al., 2014).

In addition, GSK3s have been early documented to affect the activity of transcription factors or cofactors. For example, phosphorylation of auxin response factors ARF7 and ARF19 by BIN2 suppresses their interaction with the AUX/IAAs repressors and enhances auxin signaling export in enhancing lateral root development (Cho et al., 2014). AtSK11 and AtSK12 phosphorylate the WD40 motif-containing transcriptional cofactor transparent testa glabra 1 (TTG1), influencing the interaction between TTG1 and a MYB domain transcription factor transparent testa 2 (TT2), which eventually influence the carbon partitioning between various parts of developing seed (Li et al., 2018). GSK3s also affect the DNA-binding ability or transcriptional activity of transcription factors, such as BES1, BZR1, ARF2, rice growth regulating factor GRF4 (reduced), golden 2-like 1 (GLK1) and cesta CES (enhanced) (Vert and Chory, 2006; Vert et al., 2008; Khan et al., 2014; Che et al., 2015; Duan et al., 2015; Zhang et al., 2021). The transcription factors or cofactors targeted by GSK3s proteins regulate the expression of downstream plant stress responsive genes, and eventually allow GSK3s to participate in plant response to environmental stresses.

## 4 Crosstalk between the GSK3s and phytohormones

Plant hormones are essential signaling compounds in regulating the interactions between plants and their complex biotic and abiotic environments. GSK3s have been found to mediate the crosstalk between BR and other hormones including auxin, abscisic acid (ABA), jasmonic acid (JA) and salicylic acid (SA). GSK3s coordinate actions of auxin through phosphorylating the auxin transcription factors (ARFs) including ARF2, ARF5, ARF7, ARF9, and OsARF4 (Vert et al., 2008; Cho et al., 2014; Han et al., 2018; Hu et al., 2018). The involvement of GSK3s proteins in the crosstalk with auxin mainly regulates plant developmental processes. ABA is a major stress related hormone that integrates a wide range of stress signals such as cold, salinity, osmolarity, and drought (Zhu, 2016; Chen et al., 2020a). Recent studies have uncovered that there are multifaceted interactions between GSK3s and ABA. Subgroup III snf1-related kinase 2s (SnRK2s) are key positive regulators in the ABA signaling pathway (Umezawa et al., 2009). SnRK2.2 and SnRK2.3 can interact with, be phosphorylated and thus be activated by BIN2, providing significant insight into the modulation of ABA signaling by GSK3s (Cai et al., 2014). BIN2 phosphorylates and stabilizes the bZIP-type transcription factor ABI5 of the ABA signaling pathway to mediate the antagonism of ABA by BR (Hu and Yu, 2014). But its activity on BES1 phosphorylation is affected by ABA insensitive 1 (ABI1) and 2 (ABI2), which are negative regulators of ABA signaling (Wang et al., 2018a). ABI1 and ABI2 interact with and dephosphorylate BIN2, thereby forming a PP2Cs-BIN2-SnRK2s module in the ABA signaling pathway. JA is another essential stress related hormone in plants, and the jasmonate ZIM domain (JAZ) proteins are key repressors of the JA pathway. GSK3s kinase proteins interact with and promote the degradation of JAZ proteins, and the interaction is ubiquitously present in plants including *Arabidopsis*, rice and cotton (He et al., 2020; Song et al., 2021). SA is a representative plant defense hormone that plays pivotal roles in immunity and systemic acquired resistance. The TGACG motif-binding transcription factors TGAs are known to mediate SA signaling. BIN2 phosphorylates and inhibits the activity of redox-sensitive clade I TGA4 during plant response to *Pst* DC3000. BR inactivates BIN2 and promotes SA responses by inactivating BIN2 (Kim et al., 2022). Moreover, recent results indicate that SA activates BIN2 which phosphorylates TGA3, enhancing TGA3 DNA binding ability, thereby activating *PR* gene expression and promoting disease resistance in *Arabidopsis* (Han et al., 2022). The advances displayed in this section indicates that GSK3s proteins function as hubs of phytohormones signaling pathways mainly through interacting with the transcription factors of these pathways, which eventually influence the expression of downstream responsive genes of these phytohormones. The interaction between GSK3s and other key node genes of these phytohormones needs further investigation.

## 5 Involvement of the GSK3s in plant response to multiple stresses

**Figure 2:**
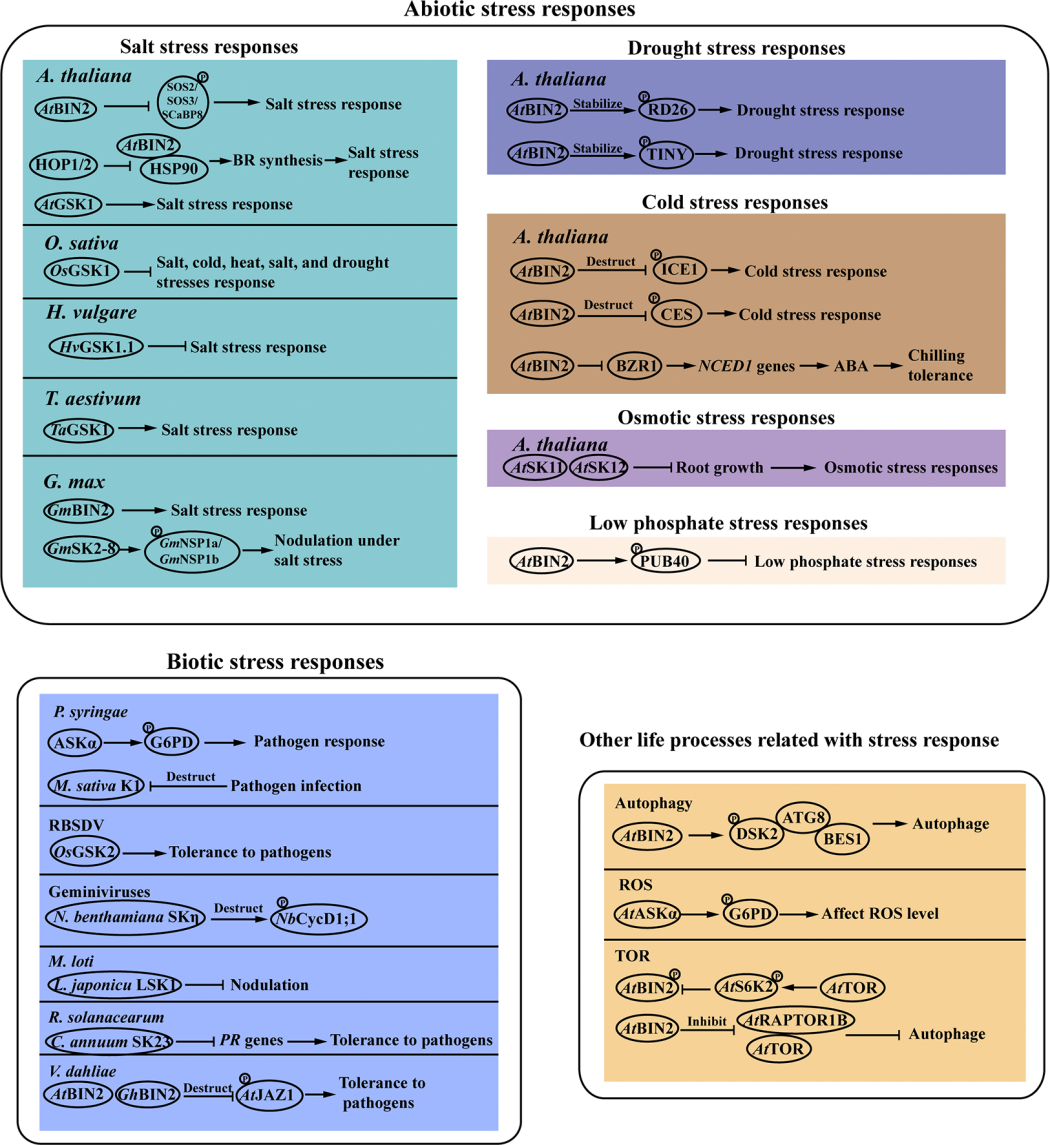
Plant stress responses mediated by GSK3-like kinases. GSK3s participated in various stress response pathways. The detailed description is given in the text. Arrows and bar ends indicate stimulatory and inhibitory action, respectively. Abbreviations: GSK3, glycogen synthase kinase 3; BIN2, brassinosteroid insentisive 2; SOS, salt overly sensitive; SCaBP8, SOS3-like calcium binding protein 8; HOP 1/2: co-chaperone heat shock protein (HSP)70-HSP90 organizing protein 1/2; HSP90, heat shock protein 90; SK2, shaggy-like kinase 2; NSP1, nodulation signaling pathway 1; RD26, NAC family transcription factor; TINY, AP2 family transcription factor; PUB40, plant U-box 40 protein; ICE1, inducer of CBF expression 1; CES, cesta; CycD1;1, Cyclin D 1.1; LSK1, shaggy -like kinase 1; DSK2, dominant suppressor of KAR 2; ROS, reactive oxygen species; G6PD, glc-6-phosphate dehydrogenase; TOR, target of rapamycin; RAPTOR1B, egulatory-associated protein of TOR 1B; PR: pathogenesis-related; JAZ: jasmonate ZIM domain; ATG8: autophagy 8; BES1: *bri1*-EMS-suppressor 1; S6K2: ribosomal protein S6 kinase 2.

Plants are challenged by various types of environmental stresses throughout their life cycles, which have profound effects on plant growth and survival. Accumulated evidence from recent findings has deciphered that GSK3s play a pivotal role in plant stress responses in model and crop species (**Figure 2**).

### 5.1 GSK3s in abiotic stresses

Soil salinization is a growing problem for agriculture worldwide. It is crucial to uncover the key components of the plant salt tolerance network. Recent studies have shown that GSK3s play important roles in regulating plant salinity stress responses. In *Arabidopsis*, overexpression of *AtGSK1* enhances plant resistance to sodium chloride (NaCl) stress, and induces the expression of some NaCl stress-responsive genes including the Ca^2+^-binding protein (*AtCP1*), the desiccation 29A (*RD29A*) and the chalcone synthase gene (*CHS1*), suggesting that *At*GSK1 is involved in the signal transduction pathway of NaCl stress responses (Piao et al., 2001). BIN2 is found to function as a molecular switch between plant salt stress response and growth through phosphorylating and affecting the activity of calcium sensors, such as SOS3, SCaBP8, and SOS2 (Li et al., 2020b). Heat shock proteins (HSPs), a group of highly conserved chaperone protein, are involved in the regulation of plant responses to salinity. HSP90 regulates the activity of the BIN2 kinase by modulating its subcellular localization (Samakovli et al., 2014). The co-chaperone heat shock protein (HSP)70-HSP90 organizing protein 1 (HOP1) and 2 (HOP2) affect HSP90-BIN2 interaction and are involved in plant salt tolerance by affecting BR signaling (Zhang et al., 2022). In rice, knockout mutants of *OsGSK1* showed enhanced tolerance to salt stress (Koh et al., 2007). Heterologous overexpression of a *GSK3* gene from soybean *GmBIN2* enhanced plant tolerance to salt in *Arabidopsis*. GSK3-like kinase *Gm*SK2-8 is strongly induced in soybean under salt stress. *Gm*SK2-8 interactes with two nodulation signaling pathway 1 (*Gm*NSP1) proteins *Gm*NSP1a and *Gm*NSP1b, which are key transcription factors involved in legume-rhizobium symbiosis. *Gm*SK2-8 phosphorylates the *Gm*NSP1a protein, and thus suppresses nodule formation under salt stress (He et al., 2021). It is reported that the GSK3 family shows altered expression in response to salt stress treatments in *Solanum tuberosum* L. Overexpression of *StSK21* provides enhanced sensitivity to salt stress in *Arabidopsis* (Huang et al., 2021). In addition, the response of the GSK3 family to salt stress is also identified in cotton (Wang et al., 2018b). In barley (*Hordeum vulgare* L.), the RNAi-mediated silencing of the target *HvGSK1.1* gene enhances the BR-dependent signaling, and generates plants with improved agricultural traits under salt stress conditions (Kloc et al., 2020). Researches show that the wheat (*Triticum aestivum* L.) *TaGSK1* gene is induced by NaCl stress, and expresses more strongly in salt-stress resistant lines than in salt-stress sensitive lines (Chen et al., 2003). From the above results we can see that some of the GSK3s proteins function as positive regulators of plant salt tolerance such as in soybean and wheat, while the GSK3s proteins reported in rice, potato, barley negatively regulate plant salt tolerance. In addition, different members in the *Arabidopsis* and cotton GSK3s family proteins show different response to the salt treatment. These results indicate that molecular mechanism of the GSK3s proteins in response to salt stress in different species or different members in the protein family is specific.

Drought stress causes a decline in the quantity of crop yields, and has become more accentuated recently due to climatic change. The GSK3 proteins are involved in drought stress tolerance. The stress-responsive NAC transcription factor RD26 participates in the interaction between growth and drought stress signaling by the phosphorylation of BIN2. BIN2 directly interacted with and phosphorylated RD26, which is required for RD26 transcriptional activation on drought-responsive genes (Jiang et al., 2019). *Arabidopsis* stress-inducible AP2/ERF transcription factor TINY positively regulates drought responses by activating drought-responsive genes. The BR negative regulator BIN2 phosphorylates and stabilizes TINY, which provides a mechanism for BR-mediated down-regulation of TINY to prevent activation of stress responses (Xie et al., 2019).

GSK3s are also involved in osmotic, low temperature and low phosphate stresses. AtSK11 and AtSK12 are involved in the mild osmotic stress (-0.4 MPa) response in *Arabidopsis thaliana*. They negatively regulate the induction of root growth in response to mild osmotic stress (Dong et al., 2020). Plant U-box 40 protein (PUB40) is a ubiquitin E3 ligase and mediates the proteasomal degradation of BZR1 in *Arabidopsis*. The interaction between PUB40 and BZR1 influences root tolerance to the low phosphate stress. BIN2 phosphorylates and stabilizes PUB40 to promote BZR1 degradation and enhances the interaction between PUB40 and BZR1, thus reducing plant tolerance to the low phosphate stress (Kim et al., 2019). Moreover, BIN2 is also regulated by the E3 ubiquitin ligase KIB1 as is mentioned above (Zhu et al., 2017). These results indicate that the E3 ubiquitin ligases play pivotal roles in the BIN2-mediated regulation of BR signaling pathway transduction. BR signaling and downstream transcriptional cascades are reported to be involved in regulating plant cold tolerance (Khan et al., 2014; Eremina et al., 2016; Li et al., 2017). As repressors of BR signaling, BIN2 and its homologs mediate the phosphorylation of BZR1 and inducer of CBF expression 1 (ICE1) to facilitate their degradation, and thus affect downstream transcriptional cascades related to cold stress response (Ye et al., 2019). BIN2 negatively regulates chilling tolerance in tomato. BIN2 also regulates the accumulation of BZR1, which controls the expression of ABA biosynthesis gene 9-cis-epoxycarotenoid dioxygenase 1 (*NCED1*) in tomato. These results demonstrate that BR signaling positively regulates chilling tolerance *via* ABA biosynthesis in tomato (An et al., 2023). Moreover, cesta (CES) is a bHLH transcription factor of BR signaling and affects plant cold tolerance (Eremina et al., 2016). CES degradation is also promoted by BIN2-mediated phosphorylation, but whether and how this protein turnover is associated *in vivo* with cold stress response should be further explored.

### 5.2 GSK3s in biotic stresses

Biotic stresses including bacteria, fungi, oomycetes, viruses and insects, wreak havoc on agricultural products worldwide and increase the risk of starvation in many areas. Advancing researches have shown that GSK3s play multifaceted roles in plant responses to various kinds of biotic stresses and is tightly regulated during plant response to pathogen infection (Wrzaczek et al., 2007). It is reported that the GSK3-like kinase ASKα is identified as a positive regulator of plant immune signaling (Stampfl et al., 2016). Loss of *ASKα* attenuates, whereas its overexpression enhances, diverse pattern-triggered immunity (PTI) responses, which is the first layer of plant immunity against pathogenic microbes. The bacterial pathogen *Pseudomonas syringae* glucose-6-phosphate dehydrogenase (G6PD) is the key enzyme of the oxidative pentose phosphate pathway and is phosphorylated by ASKα. Rice black-streaked dwarf virus (RBSDV), a double-stranded RNA virus, causes acute growth abnormalities in plants and results in serious yield losses in cereal crops. Plants over-expressing *OsGSK2* display milder symptoms than the control, suggesting a positive role of *Os*GSK2 in suppressing RBSDV infection in rice (He et al., 2020). Geminiviruses transmitted by whiteflies cause severe developmental abnormalities in plants. The C4 protein encoded by geminiviruses induces abnormal cell division that determines viral symptoms. The tomato leaf curl Yunnan virus (TLCYnV) C4 protein interacts with and affects the subcellular localization of *Nicotiana benthamiana* GSK3 protein *Nb*SKη, eventually impairing GSK3-mediated degradation of cell division associated protein *Nb*CycD1;1 (Mei et al., 2018; Mei et al., 2020). In *Medicago sativa*, the GSK3-like kinase *Ms*K1 is important for innate immunity and limits the severity of infection caused by virulent bacterial pathogen *P. syringae*. *Ms*K1 activity is downregulated by the elicitor cellulase. Cellulase treatment also triggeres the degradation of the *Ms*K1 protein (Wrzaczek et al., 2007). In *Lotus japonicu*, nodulation is mainly formed through the symbiotic nitrogen-fixing bacterium *Mesorhizobium loti* infection. Studies show that lotus SHAGGY-like kinase 1 (LSK1) is required to suppress nodulation (Garagounis et al., 2019; Solovou et al., 2021). *Ralstonia solanacearum* is a devastating soil-borne bacterium that causes wilting disease in over 200 economically-important plant species. The GSK3/SHAGGY-like kinase *Ca*SK23 negatively regulates plant response to *R. solanacearum* attack in *Capsicum annuum* (Qiu et al., 2018). We also find that BIN2 negatively regulates plant defence against *Verticillium dahliae* in *Arabidopsis* and cotton, consistent with others reports on the regulatory function of GSK3s proteins (Song et al., 2021).

### 5.3 GSK3s in other life processes related to stress response

Autophagy is a highly conserved quality control mechanism in which harmful or unwanted cellular components are delivered into lytic vacuoles for recycling, and can promote plant resistance to various stresses (Signorelli et al., 2019; Yang et al., 2020). The GSK3-like kinase is one of the key regulators of autophagy (Nolan et al., 2017). The dominant suppressor of KAR 2 (DSK2) is a ubiquitin receptor protein that targets BES1 to the autophagy pathway by interacting with ATG8, a ubiquitin-like protein that directs autophagosome formation and cargo recruitment. BIN2 can phosphorylate DSK2 and promote DSK2-ATG8 interaction, which ultimately targets BES1 for degradation (Nolan et al., 2017). Reactive oxygen species (ROS) play a key role in the acclimation process of plants to abiotic and biotic stresses (Choudhury et al., 2017). Researchers have found that the interaction of BIN2 and BES1 is dependent on oxygen, which can directly modify BIN2 (Song et al., 2019). In *Arabidopsis*, *At*ASKα regulates plant stress tolerance by activating glc-6-phosphate dehydrogenase (G6PD) responsible for maintaining cellular redox balance. Plants overexpressing *ASKα* have low levels of ROS in stress responses and are more tolerant to salt stress (Dal Santo et al., 2012; Stampfl et al., 2016). The target of rapamycin (TOR) is an atypical Ser/Thr protein kinase that is evolutionally conserved among yeasts, plants, and mammals. TOR signaling is involved in plant adaptation to nutrient deficiency and various abiotic stresses (Fu et al., 2020). The TOR downstream effector S6K2 can phosphorylate BIN2 protein, suggesting that BIN2 acts as a downstream effector of TOR signaling (Xiong et al., 2017). The egulatory-associated protein of TOR 1B (RAPTOR1B) is an important component of plant TOR complex. In the absence of BR, the BIN2 kinase directly phosphorylates and inhibits the activity of RAPTOR1B. Furthermore, autophagy is negatively regulated by TOR. Phosphorylation of RAPTOR1B by BIN2 thus activates the autophagy pathway. In the presence of BR, the inhibition effect of BIN2 on RAPTOR1B is attenuated, and results in increased TOR activity and ATG13a phosphorylation, and decreased autophagy activity (Liao et al., 2022). These results suggest that BIN2 functions as a key hub in the crosstalk between TOR and BR signaling pathways. Further studies are required to fully elucidate whether and how BIN2 interacts with other components of TOR signaling pathway.

## 6 Conclusion

The glycogen synthase kinase 3 protein was first identified in animals, and functions as an ancestral kinase of the stress response in eukaryotes. With the identification of additional proteins and other novel substrates that regulate their functions, our knowledge of plant GSK3s has inceased significantly over the last decades. It is now clear that the GSK3 proteins are involved in numerous stress-response pathways that influence animal health and plant reproduction. In animals, the GSK3s are activated under stress conditions, and determine cell fate based on its subcellular localization and specific partners (Racaud-Sultan and Vergnolle, 2021). GSK3s are found to be activated in pathologies such as inflammation and cancer, where adult stem cells are downregulated (Murata, 2018). Stem cells control tissue regeneration throughout the life of organisms, and should be protected from stressors. The microenvironment of adult stem cells is named “niche”. Numerous studies have shown that the GSK3s proteins are found to function as a sensor of the adult stem cell niche. Besides, the GSK3s are nodes of signaling pathways controlling survival, proliferation, adhesion and differentiation in adult stem cells (Racaud-Sultan and Vergnolle, 2021). Plant stem cells reside within the meristems and are also defined by their ability to self-renew and to generate new tissues (Heidstra and Sabatini, 2014). During plant growth, procambial and cambial cells in the vascular system self-proliferate and differentiate into xylem cells, and are mainly regulated by a peptide ligand and its receptor; tracheary element differentiation inhibitory factor (TDIF) and TDIF receptor (TDR) (Ito et al., 2006). Plant GSK3 proteins are crucial downstream components of TDIF–TDR signaling and regulate xylem cell differentiation (Kondo et al., 2014).

Although our understanding of GSK3s is advanced, many fundamental questions related to the gene family and its associated proteins remain unknown. For example, plants contain divergent GSK3-like kinases, but our current knowledge of the proteins comes mainly from subgroups I and II from a limited number of species. It requires understanding how GSK3s in other subgroups (e.g., III and IV) and species and what other regulatory substrates are involved in plant response to diverse environmental stimuli. The subgroups III and IV GSK3-like kinases and substrates of GSK3-like kinases reviewed here potentially provide new alleles to improve stress resistance in crops through engineering, which is an effective strategy for crop breeding processes. As mentioned above, GSK3s primarily affect hormonal signaling. Whether and how GSK3s directly regulate hormone biosynthesis is unclear and needs to be addressed. Such knowledge gap can be effectively bridged by applying recently emerging technologies such as genome-editing systems, single cell transcriptomics and deep tissue proteomics for research. Deciphering these molecular mechanisms controlling GSK3s function in detail will contribute to a better understanding of how internal and external signals integrate and branch for plant adaptation to the environment, which can eventually be transferred to stress breeding to ensure food production and ecological sustainability.

## Author contributions

MR, JsZ, YSo and YW conceived and planned this review paper. YSo and YW prepared and drafted the manuscript. JsZ, QY, YSu and JlZ revised the manuscript. All authors contributed to the article and approved the submitted version. All authors contributed to the article and approved the submitted version.
